# Adapting and optimizing GCaMP8f for use in *Caenorhabditis elegans*

**DOI:** 10.1093/genetics/iyae125

**Published:** 2024-07-29

**Authors:** Jun Liu, Elsa Bonnard, Monika Scholz

**Affiliations:** Max Planck Research Group Neural Information Flow, Max Planck Institute for Neurobiology of Behavior—caesar, Bonn 53175, Germany; Max Planck Research Group Neural Information Flow, Max Planck Institute for Neurobiology of Behavior—caesar, Bonn 53175, Germany; International Max Planck Research School for Brain and Behavior, Bonn 53175, Germany; Max Planck Research Group Neural Information Flow, Max Planck Institute for Neurobiology of Behavior—caesar, Bonn 53175, Germany

**Keywords:** genetically encoded indicator, neuronal activity, calcium imaging, pharyngeal muscle, GCaMP

## Abstract

Improved genetically encoded calcium indicators (GECIs) are essential for capturing intracellular dynamics of both muscle and neurons. A novel set of GECIs with ultrafast kinetics and high sensitivity was recently reported by Zhang *et al*. (2023). While these indicators, called jGCaMP8, were demonstrated to work in *Drosophila* and mice, data for *Caenorhabditis elegans* were not reported. Here, we present an optimized construct for *C. elegans* and use this to generate several strains expressing GCaMP8f (fast variant of the indicator). Utilizing the *myo-2* promoter, we compare pharyngeal muscle activity measured with GCaMP7f and GCaMP8f and find that GCaMP8f is brighter upon binding to calcium, shows faster kinetics, and is not disruptive to the intrinsic contraction dynamics of the pharynx. Additionally, we validate its application for detecting neuronal activity in touch receptor neurons which reveals robust calcium transients even at small stimulus amplitudes. As such, we establish GCaMP8f as a potent tool for *C. elegans* research which is capable of extracting fast calcium dynamics at very low magnifications across multiple cell types.

## Graphical Abstract

**Graphical Abstract:**
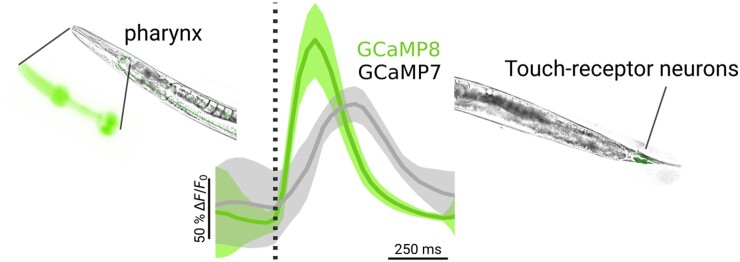

## Introduction

Genetically encoded calcium indicators (GECIs) have been vital for studying the activity of many cell types including neurons (Inoue 2021), astrocytes (Lohr *et al.* 2021), and muscles (Kerr *et al.* 2000; Collins and Koelle 2013; Collins *et al.* 2016; Sanders *et al.* 2017), as well as their coordinated activity across the heart (Salgado-Almario *et al.* 2020) and whole brain (Ahrens and Engert 2015; Kato *et al.* 2015; Nguyen *et al.* 2016; Venkatachalam *et al.* 2016; Aimon *et al.* 2019; Hallinen *et al.* 2021; Markicevic *et al.* 2021; Atanas et al. 2023) using optical imaging. Owing to this versatility, the past decades have seen rapid advancements in the brightness, photostability, sensitivity, and binding kinetics of these indicators (Dana *et al.* 2019; Zhang *et al.* 2023), enabling experiments at faster timescales (Voleti *et al.* 2019) and with less disruption to normal animal physiology. A recent paper by Zhang *et al.* (2023) described a new generation of GECIs, derived from a prior generation of GECIs (GCaMP6) through structure-guided mutagenesis. Named jGCaMP8, these indicators were demonstrated to have faster kinetics, to be brighter upon binding to Ca^2+^ and more sensitive to smaller amounts of intracellular calcium. While the study demonstrated these properties using recordings in the fruit fly, mouse, and neuronal cell culture, its use in *Caenorhabditis elegans* was not demonstrated.

In *C. elegans*, GCaMP usage has been widespread and enabled discoveries about stimulus encoding of sensory neurons using single-cell imaging (Clark *et al.* 2006; Kato *et al.* 2014; Larsch *et al.* 2015; Itskovits *et al.* 2018; Katta *et al.* 2019) and multineuron imaging in chemosensory cells (Itskovits *et al.* 2018; Pritz *et al.* 2023). In addition, GCaMP was essential for detecting the compartment-specific activity in interneurons (Hendricks *et al.* 2012) and global effects of single neurons on activity and sleep (Turek *et al.* 2013). Going beyond single or few neurons, whole-brain labeling even enabled the discovery of brain-wide coding of behavior in moving and restrained animals (Kato *et al.* 2015; Nguyen *et al.* 2016; Venkatachalam *et al.* 2016; Hallinen *et al.* 2021; Atanas et al. 2023).

With faster imaging technologies such as lightfield or lightsheet imaging (Voleti *et al.* 2019; Zhu *et al.* 2021) allowing brain imaging at up to 26 volumes per second (VPS), the true limitation for measurement is frequently the brightness and dynamic range of the indicator rather than the speed of acquisition (Kato *et al.* 2015; Hallinen *et al.* 2021; Atanas et al. 2023). Accordingly, faster and brighter indicators would positively impact these measurements in 2 ways, as they could either enable imaging of neurons driving faster behaviors, such as head swings, pharyngeal pumping, and the activity of the spiking motoneurons (Gao and Zhen 2011; Hendricks *et al.* 2012; Trojanowski *et al.* 2016; Atanas et al. 2023), or alternatively enable longer measurements due to the lower photobleaching expected. We therefore adapted the published GCaMP8f for use in *C. elegans* and measured its in vivo performance. The authors provided multiple variants with jGCaMP8f (fastest), jGCaMP8m (medium speed, more sensitive), and jGCaMP8s (lowest speed, highest sensitivity). Here, the fast variant jGCaMP8f was tested.

## Methods

### Preparing imaging plates

For imaging pharyngeal muscle activity, imaging plates were prepared similar to standard nematode growth medium (NGM) plates, but without cholesterol, and agarose is used instead of agar to reduce autofluorescence.

For imaging the touch receptor neuron (TRN) activity, an imaging chamber was prepared by filling a copper ring window (5 × 7 mm) by 2% agarose placed on an NGM plate. For ensuring the cohesion with the NGM block during the transfer to the stimulation apparatus, an extra 2% agarose on the copper ring edge was added.

### Imaging pharyngeal muscle activity at low resolution

Thirty adult animals were picked onto an imaging plate seeded with 150 µL of OP50 from an overnight culture. Animals were imaged on a commercial upright epifluorescence microscope (Axio Zoom V16; Zeiss) equipped with a 1× objective (PlanNeoFluar Z 1.0×/N.A. 0.25) with a camera sensor (acA3088-57 µm; BASLER) using a camera adapter with an additional 0.5× magnification (60 N-C ⅔″ 0.5×; Zeiss) at 16× nominal (0.5× on camera) magnification resulting in a field of view of 1.5 cm × 1.0 cm. Videos of the lawn were recorded for 10 min at 30 frames per second (fps). GCaMP7f images were recorded with a camera gain of 25 dB, and GCaMP8f had to be reduced to a gain of 19 dB to avoid overexposure.

### Imaging pharyngeal muscle activity at high resolution

Adult animals were loaded into microfluidic chips designed for measuring pharyngeal pumping (Scholz *et al.* 2016; Lee *et al.* 2017). The microfluidic chamber was perfused with 10 mM 5-HT prepared with RediPrep serotonin powder (InVivo Biosystems, USA). After 10 min, most animals showed regular pharyngeal pumping. Animals remained immersed in 5-HT during the recordings. The preparation was imaged on a commercial upright epifluorescence microscope (Axio Zoom V16; Zeiss) equipped with a 1× objective (PlanNeoFluar Z 1.0×/N.A. 0.25) with a camera sensor (acA3088-57 µm; Basler) using a camera adapter with an additional 0.5× magnification (60 N-C ⅔″ 0.5×; Zeiss) at 180× nominal (5.626× on camera) magnification. The frame rate was 30 fps with 30 ms exposure time. The resulting images were cropped to a region containing the entire pharynx of the worm using Python and rotated for display. The relative fluorescence $\Delta F/F_{0}$ was calculated from the mean intensity across the entire image relative to the baseline (5th percentile of the data). Peaks were detected using python and aligned to their respective maximum. The height of the peak and the half-decay time were extracted for each peak. Samples were selected for similar mean pumping rates and lower mean rates to allow for stable baselines to develop between peaks.

### Imaging and analysis of pumping rates from brightfield images

Animals were imaged on a dissection microscope (Axio Zoom V16; Zeiss) at 125× nominal (0.614 µm/px) resolution with a white light-emitting diode (LED) light source. Videos were recorded for 30 s at 30 fps. Animals were manually kept centered in the field of view. Three hundred frames of data (corresponding to 10 s) that had a clearly visible grinder were counted by visualizing the data in Fiji (Schindelin *et al.* 2012). Pumping rates were calculated as pumping events/10 s.

### Analysis of pharyngeal pumping rates in fluorescence images

Images were analyzed using PharaGlow (Bonnard *et al.* 2022). The peaks of the resulting intensity traces were found using the built-in peak detection, and an average pumping rate per tracklet was calculated as *N*_pumps_/duration.

### Confocal imaging of the TRNs

Young adults expressing GCaMP7f or GCaMP8f and mKate2 were immobilized in 50 mM sodium azide and mounted on a 2% agarose pad on a microscope slide under a cover slip. They were imaged using a Leica Stellaris confocal microscope (Leica Microsystems, Germany) equipped with a 25×/0.95 NA water immersion objective lens. Fluorophores were excited using laser lines at 479 nm for GCaMP7f/8f and 589 nm for mKate2, and hybrid detectors were used for signal detection. Image acquisition was conducted using LAS X software (Leica Microsystems, Germany) and processing using Fiji (Schindelin *et al*. 2012).

### Delivering touch stimuli

Substrate vibrations providing a gentle touch stimulus were delivered by gluing a 48 mm diameter piezo buzzer (APS4812B-LW100-R, PUI Audio Inc., USA) on a 60 mm diameter Petri dish housing the imaging chamber. The piezo element was driven by a 1 s 630 Hz sinusoidal voltage at various amplitudes (0, 2.5, 5, 10, 15, and 20 volts peak to peak [Vpp]). The stimulus delivery and the synchronization with the camera acquisition were controlled in a customized program (LabVIEW, National Instruments, USA) using a data acquisition card (BNC-2090A, National Instrument).

### Imaging TRN activity

Three to six adult animals were picked onto the imaging chamber and immobilized in 10 mM levamisole (Sigma-Aldrich). The animals were imaged using an epifluorescence microscope for ratiometric calcium imaging equipped with a 20× objective (S Plan Fluor LWD, N.A. 0.70, Nikon). The resulting field of view was 250 × 500 µm with a pixel size of 0.24 µm per pixel.

To excite GCaMP7f/8f and mKate2 fluorophores, the cyan (470/24 nm, Chroma) and green (575/15 nm, Semrock) lines from an LED lamp (Spectra X light engine, Lumencor) were projected onto the sample. Transmitted and emitted light were filtered using a triple-edge dichroic beamsplitter (409/493/596 nm, Semrock). To simultaneously image GCaMP8f and mKate2, a dual view with a 585 nm beamsplitter (DV2, Photometrics) was used. Each channel was projected onto a half of an sCMOS camera (Zyla, Andor) at 30 Hz acquisition rate with an exposure time of 33 ms and a 16 bit readout depth. Animals were acclimated to blue light under the microscope for 20 s before being exposed to stimuli of increasing intensity. Each recording consisted of a single stimulus exposure, including a 20 s prestimulus period and a 60 s poststimulus period.

### Ratiometric analysis

The resulting images were automatically split into individual channel images and registered for an optimal overlay using MATLAB. Using the mKate2 channel images, manually defined regions around each posterior lateral microtubule (PLM) neuron pair including background were cropped in Fiji. Within each region, the neurons were tracked using the Fiji plugin TrackMate (Tinevez *et al.* 2017) defining a circular region of interest (ROI) around the neuron.

Fluorescent signals were calculated as the 95 percentile of the intensity in the circular ROI using Python. Similarly, the local background was calculated as the 5th percentile and subtracted from the signal, and the resulting curves were bleaching-corrected by dividing by the overall signal decay as described by a fit with a single exponential. Then, the fluorescence for each channel was normalized relative to the baseline as follows:


$$r_{\mathrm{green}/\mathrm{red}}=\;F_{\mathrm{green}/\mathrm{red}}/F_{\mathrm{green}/\mathrm{red}}^{0}$$

where *F*^0^ is the time-averaged signal 5 s before the stimulus onset. The percentage of ratio change was calculated as follows:


$$\Delta R/R_{0}=(R-R_{0})/R_{0}\;*\;100$$

where *R* is the ratio between the normalized fluorescence of GCaMP7f/8f and mKate2 such as *R* = *r*_green_/*r*_red_ and *R*_0_ is the baseline ratio calculated from the time-averaged signals 5 s before the stimulus onset.

### Reagents

#### Plasmids

pLJ50 was made by replacing the *unc-31p* from pRL231 (*unc-31p::GCaMP7f::unc-54 3′utr*, gift of Manuel Zimmer) with *myo-2p*. pLJ52 was adapted from the sequence described in Zhang *et al.* (2023) by codon optimizing the GCaMP8f and adding 3 synthetic introns as described in Redemann *et al.* (2011). The sequence was created using the tool at https://worm.mpi-cbg.de/codons/cgi-bin/optimize.py and synthesized by GenScript. Then, GCaMP8f was subcloned into pLJ50 to replace GCaMP7f to make pLJ53 (*myo-2p::GCaMP8f::unc-54 3′utr*).

“SL2::mKate2::let-858_3′UTR” was used to replace the *unc-54 3′UTR* in pLJ53 to make pLJ54 (*myo-2p_GCaMP8f_SL2_mKate2_let-858_3′UTR*). Then, *myo-2p* was further replaced by *mec-17p* to make pLJ57 (*mec-17p_GCaMP8f_SL2_mKate2_let-858_3′UTR*). GCaMP8f from pLJ57 was replaced by GCaMP7f to make pLJ80 (*mec-17p_GCaMP7f_SL2_mKate2_let-858_3′UTR*).

## Results

To determine whether GCaMP8f also shows the reported improved properties as a fluorophore in *C. elegans*, we wanted to compare it with the prior generation of fluorophores in a standardized setting. GECIs are frequently compared in spiking neurons with stereotyped action potentials, which simplifies the analysis: as the underlying shape is expected to remain the same, any change in the signals readout by imaging can be attributed to changes in the indicator properties. However, as *C. elegans* neuronal activity is characterized predominantly by graded potentials (Goodman *et al.* 1998), we decided to instead use the stereotypical contractions of the pharynx to test the properties of this GECI. *C. elegans* uses its pharynx to ingest bacteria, and this muscle shows action potentials shaped by voltage-gated calcium and potassium channels (Shtonda and Avery 2005). The quasi-rhythmic action of the pharyngeal muscle was also the first behavior whose activity was visualized with a GECI in *C. elegans* [i.e. Cameleon (Kerr *et al.* 2000)].

**Fig. 1.**
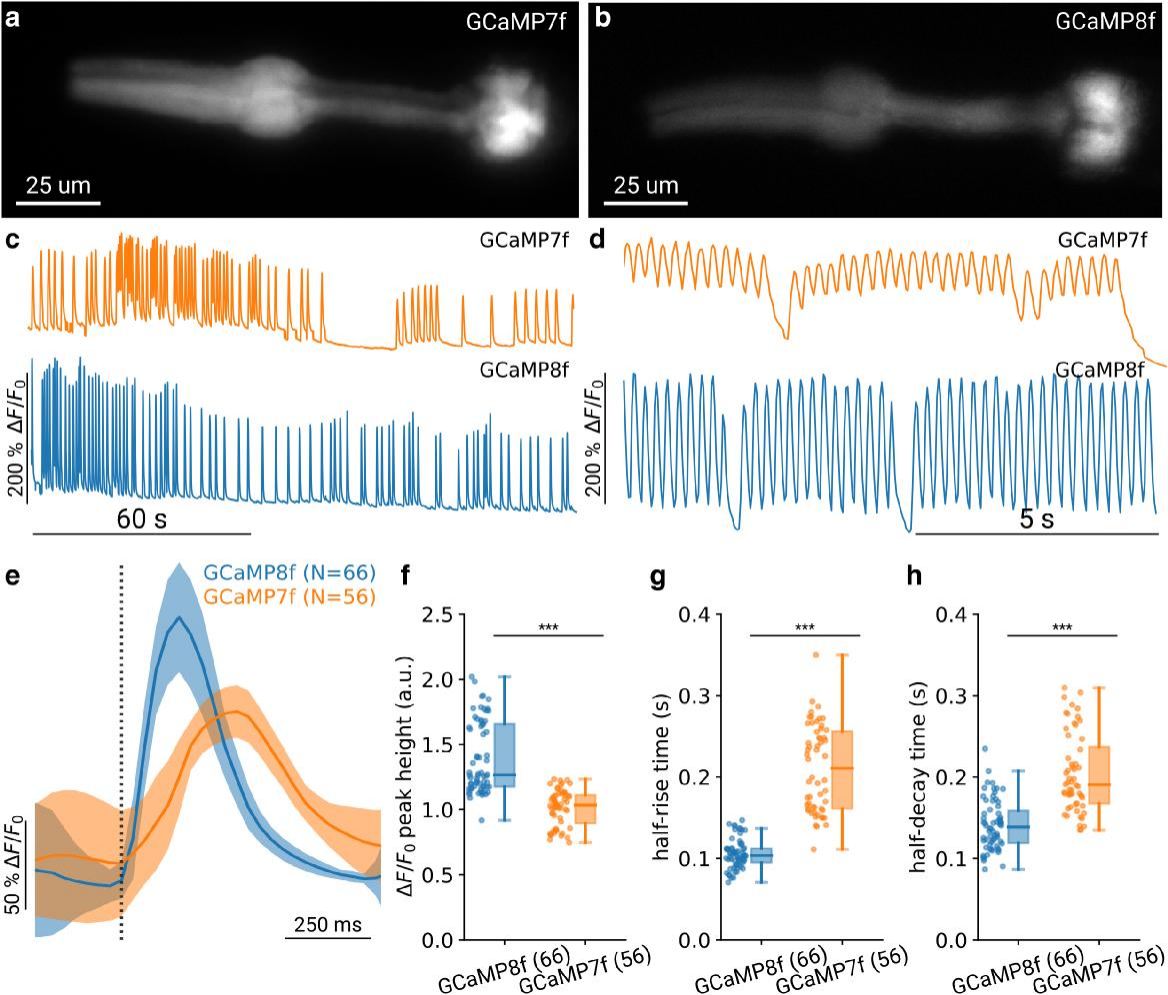
Improved detection of calcium dynamics with GCaMP8f due to faster indicator kinetics. Images showing a) GCaMP7f and b) GCaMP8f expressed under the *myo-2* promoter in adult *C. elegans* during pharyngeal pumping. c) Example traces of pharyngeal muscle activity with GCaMP7f (top) and GCaMP8f (bottom) under stimulation with 10 mM 5-HT for a representative animal during slower overall pumping and d) for faster pumping animals. e) Mean peak shape aligned to the onset for GCaMP7f and GCaMP8f. Shaded area indicates the standard deviation. Note that the increase in standard deviation before and after the peak is due to nearby peaks starting within the window of alignment and does not indicate an issue with detection. f) Maximal peak height for GCaMP7f (*N* = 56) and GCaMP8f (*N* = 66), respectively. g) Half-rise time for each peak, as estimated from the first moment the traces reached 0.5*maximal height. h) Half-decay time for each peak, as estimated from the first moment the traces decayed to 0.5*maximal height. *** indicates *P* < 0.001. Significance was assessed using Welch's unequal variance *t*-test.

To test the *C. elegans* optimized GCaMP8f, we used a similar approach and expressed the fluorophore in the pharyngeal muscle using the *myo-2* promoter. Based on the published sequence in Zhang *et al.* (2023), we generated a codon-optimized version for *C. elegans*, with 3 introns added, that were suggested to improve expression (Okkema *et al.* 1993). The resulting sequence was cloned into a plasmid containing the *myo-2* promoter specific to pharyngeal muscle and an *unc-54* 3′ UTR (Table 1). We then compared the activity of GCaMP8f with the previous generation of GECI, GCaMP7f (Dana *et al.* 2019) (Fig. 1a and b). By applying serotonin (5-HT), action potentials in the pharynx can be stimulated and the activity of the GECI visualized using fluorescence microscopy with limited intrinsic behavioral variability (Fig. 1c and d). We find that as described for *Drosophila melanogaster*, mouse and in neuronal cell cultures (Zhang *et al.* 2023), the indicator has a larger dynamic range (Fig. 1e and f) and shows faster kinetics compared to GCaMP7f (Fig. 1g and h). In particular, for rapid contractions (Fig. 1d), the slower indicator GCaMP7f is unable to return to the baseline between contractions, thus reducing the peak amplitude and potentially hindering detection of calcium transients.

**Table 1.** List of strains.

Strain name	Genotype	Short name in article	Source
INF491	*nonEx105[myo-2p::GCaMP7f]*	GCaMP7f	This study. pLJ50 was injected into INF125 *nonIs4[lgc-8p::NLS::cGAL(DBD):: cGAL(AD)::let-858 3′UTR + coel::RFP]* at 5 ng/µL. Then, *nonIs4* was outcrossed
INF418	*nonEx106[myo-2p::GCaMP8f::unc-54 3′UTR]*	GCaMP8f	This study. pLJ53 was injected into N2 at 5 ng/µL
INF447	*nonEx133[mec-17p_GCaMP8f_SL2_mKate2_let-858_3′UTR + unc-122p::tagBFP]*	TRN::GCaMP8f	This study. pLJ57 was injected into N2 at 40 ng/µL. *unc-122p::tagBFP* (pLJ49) was used as a marker (40 ng/µL)
INF498	*nonEx158[mec-17p_GCaMP7f_SL2_mKate2_let-858_3′UTR + unc-122p::tagBFP]*	TRN::GCaMP7f	This study. pLJ80 was injected into N2 at 40 ng/µL. *unc-122p::tagBFP* (pLJ49) was used as a marker (40 ng/µL)

**Fig. 2.**
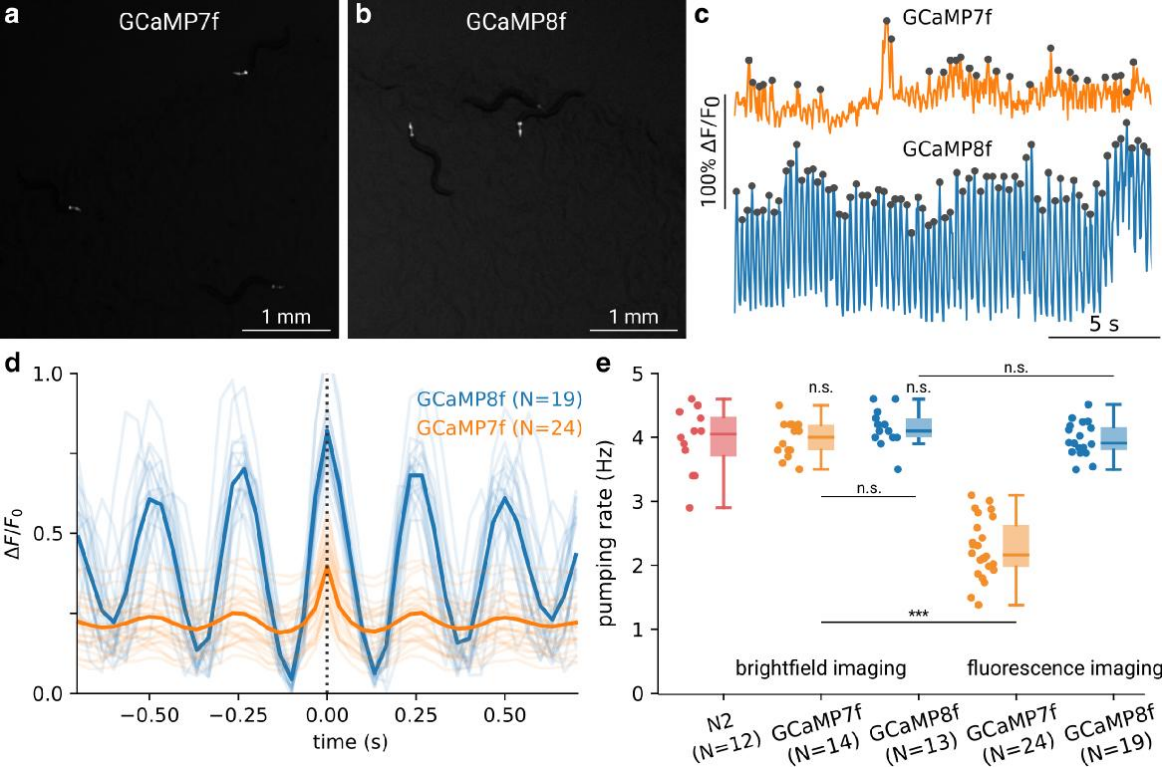
Large field-of-view measurements of pumping rates enabled by pharyngeal GCaMP8f sample region showing animals expressing a) GCaMP7f and b) GCaMP8f in the pharynx at low magnification of 0.5×. The full field of view comprises 14 mm × 10 mm. c) Examples of resulting fluorescence activity traces for GCaMP7f (top) and GCaMP8f (bottom) with peaks indicated in gray. d) Average of the detected peaks corresponding to pumping events for GCaMP7f and GCaMP8f. e) Pumping rate as measured from the peaks detected in c) and by manual counting of brightfield images at higher resolution. Note that the results for GCaMP7f are only displayed for comparison, as the lower quality of the trace yields many false detections and does not result in reliable measurements. *** indicates *P* < 0.001.Significance was assessed using a Kruskal–Wallis test followed by a post hoc Welch's unequal variance *t*-test. Multiple comparisons were corrected using the Bonferroni correction.

Given the increased dynamic range and kinetics, we wanted to test whether these properties enable new assays at lower resolution, allowing high-throughput data collection, for example by detecting the feeding contractions of the pharynx muscle. While pumping can be detected without fluorescent labels, this requires imaging at higher magnifications and often laborious postprocessing. We previously found that expressing a noncalcium-dependent fluorophore enables detecting pumping at lower magnification and in freely moving animals (Bonnard *et al.* 2022). Using animals expressing either GCaMP7f or GCaMP8f in the pharyngeal muscle, we imaged groups of worms on a plate seeded with food. Compared to our previous work (Bonnard *et al.* 2022), we could detect animals labeled with GCaMP8f and GCaMP7f at even lower magnifications (0.5× instead of 1×), enabling a field of view of 1.5 cm (Fig. 2a and b; Supplementary Fig. 1). By tracking and segmenting the signals, we could find clear, stereotyped peaks in the fluorescence signals of animals expressing GCaMP8f, but not GCaMP7f (Fig. 2c). While the brightness of GCaMP7f was sufficient to track the animals, the peaks in GCaMP7f signals were noisy, likely due to the slower indicator speeds as seen in Fig. 1d and the smaller dynamic range (Fig. 2c). We therefore concluded that the pumping rates extracted from peaks in GCaMP7f were not a reliable reflection of the true pumping rate (Fig. 2e).

One disadvantage of these indicators is their required binding of intracellular calcium. This results in sequestering some of the free calcium ions, thereby lowering their overall concentration and potentially interfering with intracellular processes. The effects of this can be observed both for GECIs that express in muscle, as well as in neurons, as strains that expressed GCaMP pan-neuronally frequently move slower than wild-type animals (Nguyen *et al.* 2016; Yemini *et al.* 2021). To estimate if calcium chelation is also detrimental to pharyngeal pumping and could impair the interpretation of such high-throughput measurements, we compared the pumping rate in brightfield images at high resolution for both strains with the N2 (wt) strain. We found that animals expressing neither GCaMP7f nor GCaMP8f showed a significantly different pumping rate from wild type, suggesting that under these expression levels, muscle function is not impaired (Fig. 2e). In addition, we found that the pumping rate measured using GCaMP8f did not differ from the rate measured using high-resolution brightfield imaging (Fig. 2e). We therefore conclude that this indicator enables robust, high-throughput analyses of feeding behavior which was not possible using GCaMP7f.

**Fig. 3.**
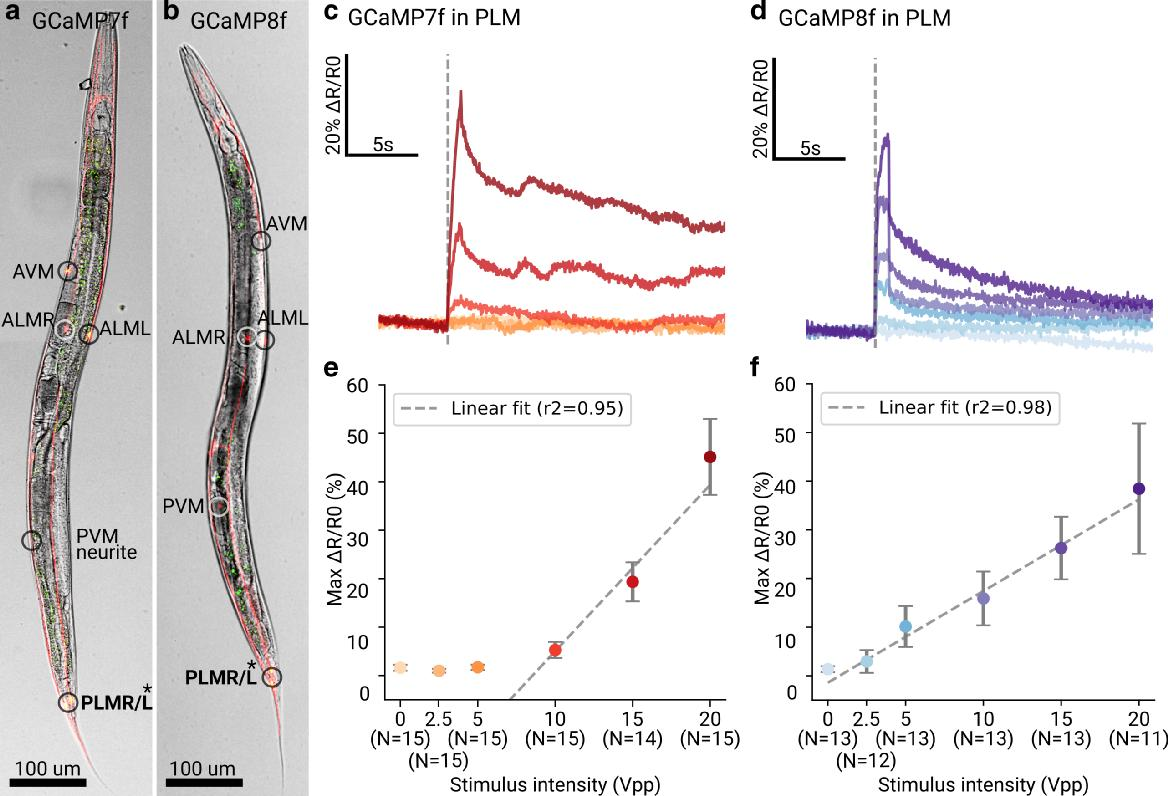
Sensitive measurement of neural activity in response to touch. a and b) Confocal microscopy images showing animals expressing GCaMP7f a) or GCaMP8f b) (green) and mKate2 (red, merged) in the TRNs. Asterisks indicate the PLM neurons among the TRNs used to extract activity. c and d) Population averaged responses of PLM neurons to graded stimulus intensities using ratiometric correction for GCaMP7f and GCaMP8f, respectively. e and f) Stimulus intensity–response curve of GCaMP7f and GCaMP8f, respectively. The response was fitted with a linear model based on prior observation of TRN linearity. Bars indicate the standard error of the mean at the maximal ratio change. The stimulus intensities are color coded in oranges for GCaMP7 and blues for GCaMP8f.

We finally wanted to test if GCaMP8f also improves functional imaging of neuronal activity. As neurons are much smaller than the pharyngeal muscle, this typically requires higher magnifications and yields noisier, lower amplitude signals compared to the data shown in Fig. 1. As TRN responses are also well characterized, they lend themselves to verify if GCaMP8f performs as expected and to check if this GECI also allows imaging at faster frame rates or lower magnifications in neuronal activity recordings. We therefore devised a protocol to stimulate the 6 TRNs (using a *mec-17* promoter). We designed a plasmid containing both a baseline fluorophore (mKate2, red, insensitive to intracellular calcium) and GCaMP8f, as well as the same construct but with GCaMP7f replacing GCaMP8f for direct comparison (Table 1). We verified the expression using confocal microscopy (Fig. 3a and b). While we could detect both mKate2 and GCaMP8f/7f in all 6 TRNs, the expression was strongest in the PLM neurons. We therefore set out to measure PLM responses to touch.

Multiple animals could be imaged simultaneously while a piezo buzzer supplied a gentle touch stimulus with varying amplitudes. To enable a fair comparison between the strains despite potentially different expression levels, we use the same exposure parameters, but adjust the excitation light intensity to match the on-camera intensity of both the red and green channels. This procedure guarantees that we do not miss calcium transients in either fluorophore due to potentially lower expression levels in either strain that would drop signals below the detection limit of the camera. By extracting the relative calcium changes, we could quantify neuronal activity dependent on stimulus amplitude (Fig. 3c–f; Supplementary Fig. 2).

While we were unable to detect PLM activity with GCaMP7f at the lowest 2 stimulus levels, we were able to detect activity across all stimuli using GCaMP8f. Albeit we may expect the sensor with higher affinity (GCaMP7f) to be more sensitive and produce brighter signals at lower stimulus intensities, here this effect is mitigated by the inherently larger brightness of the GCaMP8f-Ca^2+^ complex compared to the GCaMP7f-Ca^2+^ (Zhang *et al.* 2023). Additionally, for fast Ca^2+^ signals, the faster sensor can follow the underlying changes in intracellular Ca^2+^ concentration ([Ca^2+^]i) with higher fidelity, thus allowing it to generate more sensor-Ca^2+^ complex and thus reach higher peak intensity, before the [Ca^2+^]i decays in the cell. In contrast for long-lasting Ca^2+^ responses elicited at stronger stimulation, the concentration of the sensor-Ca^2+^ complex for the slower, high-affinity sensor (GCaMP7f) will effectively accumulate. In the extreme cases that additional Ca2+ signals are elicited, the sensor-Ca2+ complex might be unable to dissociate before [Ca2+]i rises again, resulting in apparent signal integration (Fig. 1d; see also Zhang *et al.* 2023, Extended Data Figs. 8 and 10b, c).

We observe that the activity in PLM scales linearly with stimulus amplitude (Fig. 3e and f), consistent with electrophysiological studies (O’Hagan *et al.* 2005; Eastwood *et al.* 2015; Katta *et al.* 2019) and calcium imaging data obtained with GCaMP6m (Cho *et al.* 2017). Compared to the prior study of TRNs which used an even earlier generation of fluorophore (GCaMP6m), the improved fluorophore GCaMP8f allowed a faster imaging rate (30 Hz instead of 10 Hz) and a lower magnification (20× instead of 40×), while obtaining consistent results.

## Discussion

In summary, we find that in *C. elegans* GCaMP8f is brighter upon calcium binding and shows faster rise and decay times than its immediate predecessor GCaMP7f. Accordingly, this improved fluorophore can enable new experiments studying subcellular compartments, detection of smaller transients, or allow higher throughput experiments using more animals simultaneously by imaging at low magnifications (Larsch *et al.* 2013). Importantly, as GCaMP8f also appears to have a limited impact on the nematode's intrinsic biological processes, measurements using this fluorophore will better reflect the dynamics and behavior of wild-type animals. The faster indicator kinetics and the increase in dynamic range allow imaging at faster timescales, which will enable studying neuronal transients at faster timescales for example in motoneurons (Liu *et al.* 2020). Finally, for rapid whole-brain imaging, calcium indicators need to be either expressed at high levels or conversely, very sensitive to calcium and bright to allow rapid imaging of a whole brain in 3D with short exposure times. This generation of fluorophore will likely enable new experiments unlocking the neuronal coding of both behavior and stimuli on faster timescales.

## Supplementary Material

iyae125_Supplementary_Data

## Data Availability

Strains and plasmids are available upon request for all strains and plasmids generated in this study. The plasmid maps for pLJ50, pLJ53, pLJ57, and pLJ80 are available as Supplementary Files 1, 2, 3, and 4, respectively. All data underlying the figures is available in OSF (https://osf.io/c4jrm/). Supplemental material available at GENETICS online.
