# Supplementary material for: Adapting and optimizing GCaMP8f for use in Caenorhabditis elegans
Source: Genetics. 2024 Jul 29;228(2):iyae125. doi: 10.1093/genetics/iyae125 (PMC11457936; doi:10.1093/genetics/iyae125)
Supplement: iyae125_Supplementary_Data [file iyae125_supplementary_data.zip › Supplementary_Figures_GENETICS-2024-307164.docx]

**Supplementary Figures**


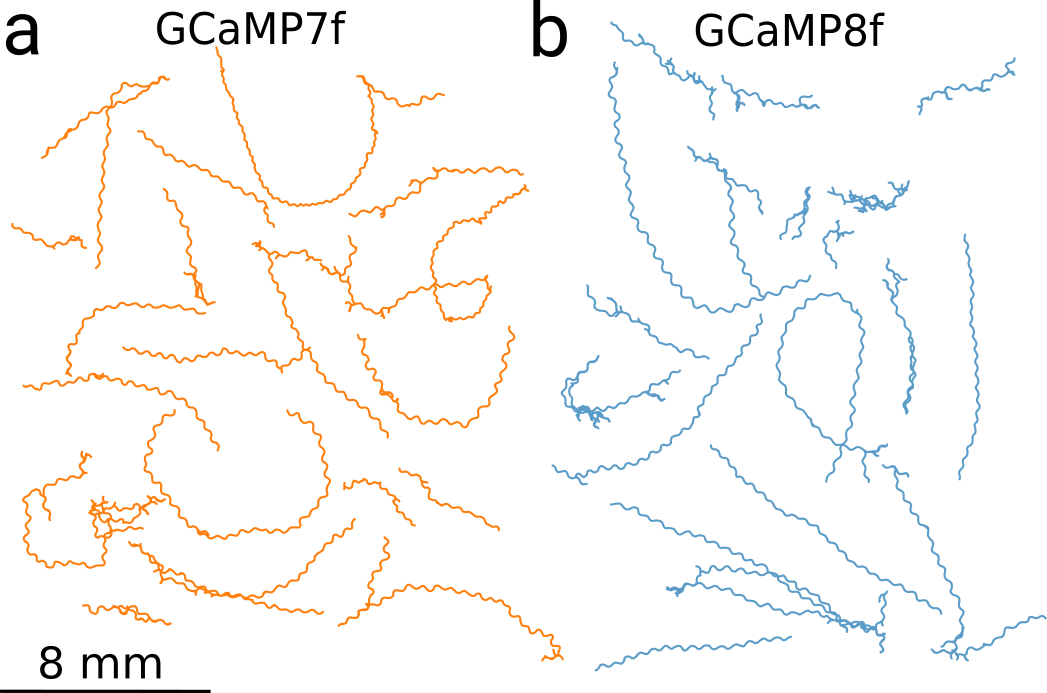


**Figure S1** Tracks obtained at 0.5x magnification for both myo-2p::GCaMP7f and myo-2p::GCaMP8f animals.


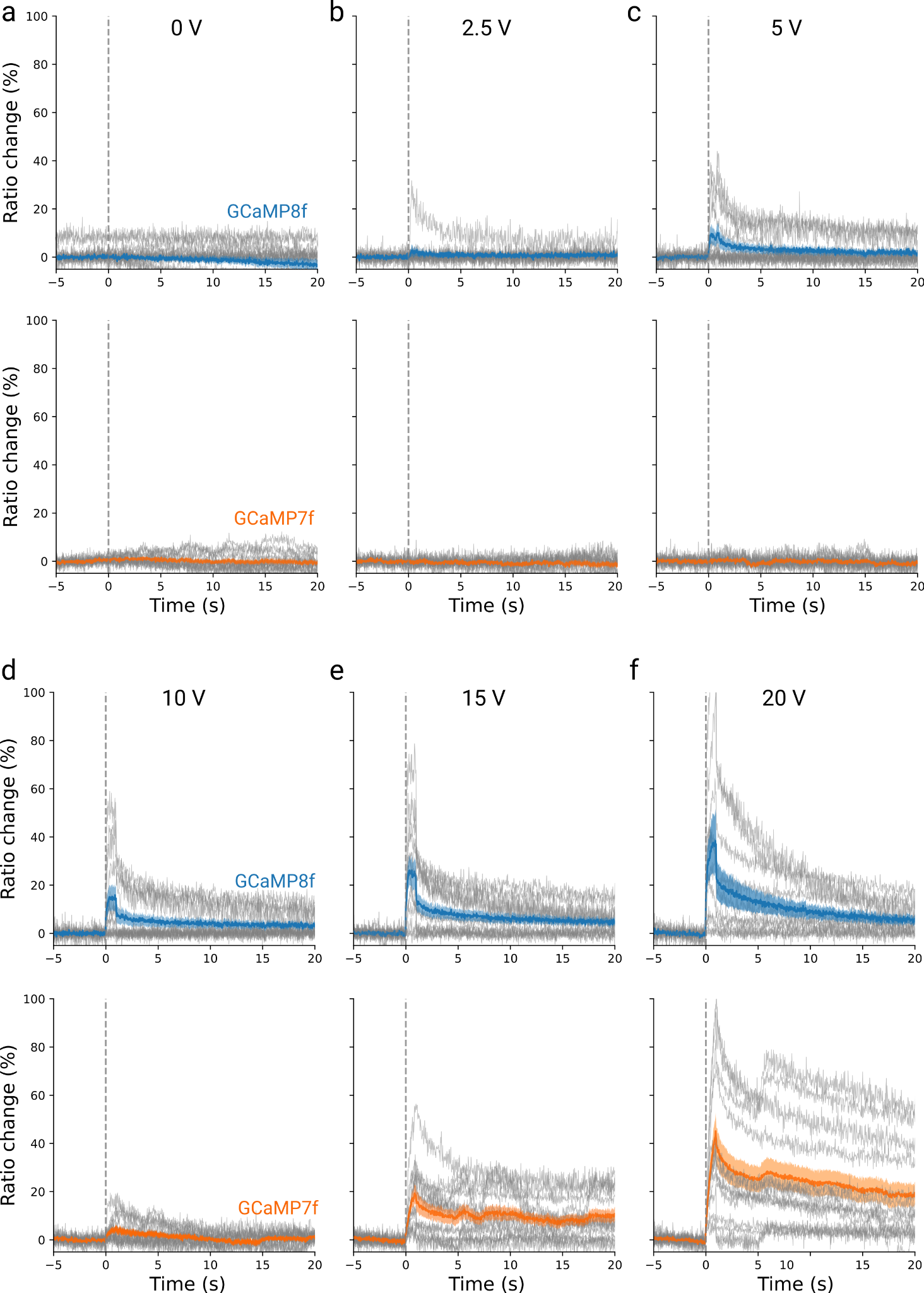

**Figure S2 Individual traces of neuronal activity as measured using GCaMP7f and GCaMP8f**(a) Traces at stimulus intensity 0 V (N=13 for GCaMP8f, N=15 for GCaMP7f) (b) 2.5V (N=12 for GCaMP8f, N=15 for GCaMP7f), (c) 5V (N=13 for GCaMP8f, N=15 for GCaMP7f), (d) 10 V (N=13 for GCaMP8f, N=15 for GCaMP7f) (e) 15 V (N=13 for GCaMP8f, N=14 for GCaMP7f) and 20V (N=11 for GCaMP8f, N=15 for GCaMP7f).
